# Expression Profiles and Clinicopathologic Features in Early Resected Non-small-cell Lung Cancer^☆^

**DOI:** 10.1016/j.ebiom.2014.10.008

**Published:** 2014-10-22

**Authors:** Rafael Rosell

**Affiliations:** Cancer Biology and Precision Medicine Program, Catalan Institute of Oncology, Hospital Germans Trias i Pujol, Badalona, Barcelona, Spain; Germans Trias i Pujol Health Science Research Institute and Hospital, Campus Can Ruti, Badalona, Barcelona, Spain; Molecular Oncology Research (MORe) Foundation, Barcelona, Spain

Lung cancer is the leading cause of cancer-related death worldwide and surgical resection is the treatment of choice for early-stage non-small-cell lung cancer (NSCLC). However, in this subgroup of patients, survival can range from 10% to 60% in stage II (with involvement of lymph nodes — N1), and stage IIIA (involvement of mediastinal lymph nodes — N2). Stage IA is the most favorable situation with very small tumors without lymph node metastasis. However, the risk of relapse is still rather unpredictable despite the many prognostic gene signatures that have been developed. In spite of the lack of commonality in many genes identified in published prognostic signatures, numerous gene expression signatures perform overlapping prognostic functions and may be able to provide uniform information on outcome in early NSCLC (Rosell et al., 2011, Chen and Chen, 2014).

To this end, Chen and Chen have reported a lung cancer prognostic index (LCPI) that includes the expression of seven genes and combines with age and stage. The LCPI permits separation of patients into three different risk groups (low, intermediate and high) following surgery. Using this LCPI, patients classified as low risk of recurrence can be deemed as not needing any further treatment. For patients deemed to be at high risk, complete surgical resection is insufficient treatment and they will require further therapy. Intriguingly, for patients in low/intermediate risk groups adjuvant chemotherapy could be detrimental for survival (Chen and Chen, 2014).

What lessons can be learned from this LCPI? NSCLC has become recognized as being a heterogeneous set of diseases (Reck et al., 2013) and, in patients with lung adenocarcinomas, epidermal growth factor receptor (EGFR) mutations are associated with response to EGFR inhibitors. Other potentially targetable oncogenes are HER2, MET, FGFR1 and KRAS as well as fusion oncogenes involving anaplastic lymphoma kinase (ALK), ROS1, neuregulin 1 (NGR1) and neurotrophic tyrosine kinase receptor 1 (NTRK1). These oncogenic lesions predict sensitivity to specific inhibitors. Potentially targetable mutations have also been identified in squamous cell carcinoma of the lung, such as discoidin domain-containing receptor 1 (DDR2), FGFR1 and others. Therefore, in most cases, adjuvant chemotherapy may not be the correct strategy. In the paper from Chen and Chen, adjuvant chemotherapy has a nefarious effect on patients with low/intermediate risk of relapse according to the LCPI (Chen and Chen, 2014). Intriguingly, according to the LCPI for patients with high risk, the effect of chemotherapy could be negligible. Two large meta-analyses of adjuvant and preoperative chemotherapy in early resected NSCLC show an absolute increase in survival of 4% at 5 years (Arriagada et al., 2010, Arriagada et al., 2014). Neither meta-analysis showed clear evidence that age, histology, and clinical stage benefited more or less from adjuvant or preoperative chemotherapy (Arriagada et al., 2010, Arriagada et al., 2014). However, in the adjuvant chemotherapy meta-analysis, there was a trend towards a negative effect of chemotherapy in the small subgroup of stage IA patients (Arriagada et al., 2010). A 14-gene expression assay using quantitative PCR was able to discriminate the risk of recurrence in stage I non-squamous small cell carcinoma with 5-year overall survival (OS) of 71.4% in low-risk, 58.3% in intermediate-risk and 49.2% in high-risk patients (Kratz et al., 2012). Genes in this prognostic signature, including BRCA1 and YAP1, are central to crucial oncogenic pathways (Rosell et al., 2011, Kratz et al., 2012). Acquired resistance to KRAS suppression in a KRAS-driven murine lung cancer model involves increased YAP1 signaling. KRAS and YAP1 converse on the transcription factor FOS and activate a transcriptional program involved in regulating the epithelial–mesenchymal transition (EMT) (Shao et al., 2012). Therefore, among the different gene signature models, there are still many inconsistencies. Although histology was not found to be relevant in the meta-analysis in early resected NSCLC (Arriagada et al., 2010), recent data indicate that using the new International Association for the Study of Lung Cancer (IASLC)/American Thoracic Society (ATS)/European Respiratory Society (ERS) classification, micropapillary- and solid-predominant adenocarcinomas have significantly worse OS, probability of freedom from recurrence and disease-specific survival than those with lipedic-, acinar- and papillary-predominant subtypes (Hung et al., 2014). In Chen and Chen's LCPI, histology was not relevant. They show OS probability at 10 years in the high-risk group to be 9.5%, while for the intermediate-risk group this was 39%, and 76% for the low-risk group. However, it could be convenient to provide data on disease-specific survival when calculating survival, since most patients are relatively elderly at time of diagnosis and can die from non-cancer-related causes.

There is a need for a cancer systems-biology approach that can help to build the optimal prognostic and predictive gene signatures. Chen and Chen have tried to resolve this challenge with the aid of ever increasing computing power. Although information regarding intergene regulatory relationships and their cascades is accumulating, the whole picture regarding gene regulatory circuitry remains elusive. Further research is to be encouraged, employing systems biology-based approaches to identify the altered regulatory circuitry involved in early NSCLC and decipher the web of interconnected genes that confer hallmark cancer capabilities (Hanahan and Weinberg, 2000). The only caveat, as Chen and Chen have already shown, is that adjuvant chemotherapy can be detrimental. Also, adjuvant therapy with EGFR tyrosine kinase inhibitors has been demonstrated to increase the risk of death with a hazard ratio of 3.1 in the subgroup of adjuvant patients with EGFR mutations (Goss et al., 2013). This can occur since EGFR inhibition can paradoxically activate other signaling pathways and therefore decrease survival in patients in whom surgical resection was performed with curative intent.
